# Determination of glyphosate and aminomethylphosphonic acid residues in Finnish soils by ultra‐high performance liquid chromatography–tandem mass spectrometry

**DOI:** 10.1016/j.mex.2023.102397

**Published:** 2023-09-23

**Authors:** Rämö Sari, Välimäki Juho, Siimes Katri, Uusi-Kämppä Jaana

**Affiliations:** a Natural Resources Institute Finland (Luke), Myllytie 1, Jokioinen 31600, Finland; b MTT Agrifood Research, Finland; c current: KVVY Tutkimus Oy, Patamäenkatu 24, Tampere 33900, Finland; d Finnish Environmental Institute (Syke), Latokartanonkaari 11, Helsinki 00790, Finland; e Natural Resources Institute Finland (Luke), Tietotie 4, Jokioinen 31600, Finland

**Keywords:** Multiple reaction monitoring, Matrix-matched, Internal standard, N-(phosphonomethyl)glycine), AMPA, Determination of glyphosate and AMPA in Finnish soil by MRM-UHPLC-MS/MS-technique

## Abstract

Glyphosate [N-(phosphonomethyl) glycine] (GLY) adsorbs strongly in Finnish soils. A new method for GLY and its main degradation product, aminomethylphosphonic acid (AMPA) residues in clay soils (Protovertic Luvisol) was developed and validated. A new method was necessary because the previous one required laborious cleaning pre-treatments, and its recovery was quite poor (<40%–70%). In the new method, the earlier method's extraction solvent, 0.1 M potassium hydroxide (KOH), was replaced by more effective 0.6 M KOH. The old post-column high-performance liquid chromatography and fluorescence (HPLC-FLD) method was replaced by the ultra-high performance liquid chromatography–tandem mass spectrometry (UHPLC-MS/MS) method. Compounds were identified as their fluorenyl methyl chloroformate (FMOC) derivatives by a multiple reaction monitoring (MRM) technique and quantified by an internal standard method utilising multipoint matrix-matched calibration. Glufosinate-ammonium (GLUF) was used to monitor the effectiveness of extraction with good recovery (80–119%). All calibration curves were found to be linear (R^2^ ≥ 0.98) in the studied calibration range (0.01–3.31 mg kg^−1^ in fresh soil). The repeatability and reproducibility were 25% and 28% for GLY, and 20% and 24% for AMPA in real research soil samples. The method was effective throughout the calibration range in all the studied Finnish agricultural soils.

• An improved method was created to analyse glyphosate (GLY) and AMPA in Finnish clay soil.
• The challenge caused by strong GLY adsorption on soil was solved by using multipoint matrix-matched calibration curve samples which were prepared identically with the research samples.
• The method performed well in all tested clay, loam and sandy loam soils.

## Graphical abstract

**Figure:**
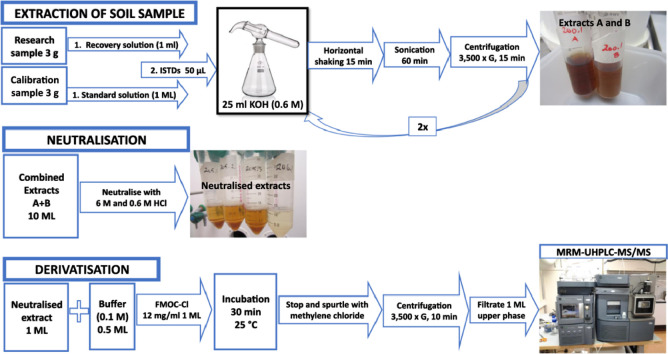

Specifications table

**Table:** 

Subject area:	Chemistry
More specific subject area:	Herbicide residue analysis
Name of your method:	Determination of glyphosate and AMPA in Finnish soil by MRM-UHPLC-MS/MS-technique
Name and reference of original methods:	**The original method:**
	Determination of glyphosate and AMPA in Finnish soil samples as their post-column OPA derivatives by liquid chromatography fluorescence detector, **published in:**
	Laitinen, P., Siimes, K., Eronen, L., Rämö, S., Welling, L., Oinonen, S., Mattsoff, L., and Ruohonen-Lehto, M. 2006. Fate of herbicides glyphosate, glufosinate-ammonium, phenmedipham, ethofumesate and metamitron in two Finnish arable soils. Pest Manag Sci 62:473–491
	**The published method, which has been utilised for new method development**:
	Ibañez, M., Pozo, O.J., Sancho, J.V., Lopez, F.J., and Hernandez, F. 2005. Residue determination of glyphosate, glufosinate, and aminomethylphosphonic acid in water and soil samples by liquid chromatography coupled with electrospray tandem mass spectrometry. Journal of Chromatography A, 1081:145–155.
Resource availability:	**Waters Acquity UPLC Xevo TQ MS** (Waters Corporation, Milford, MA, USA) was used in the identification and quantification of all compounds.
	The **Intellistart** function of the instrument was used to optimise MS/MS method parameters of the analytes.
	**Acquity™ Premier BEH C18 columns** (Waters Corporation, Milford, MA, USA) have been available since 2020. Premier means a surface modified column that increases analyte recovery, sensitivity, and reproducibility by minimising analyte/surface interactions that can lead to sample losses. The surface modification is only on the metal surfaces of the column, so it will not affect the desired retention mechanisms (personal contact by email 2 June 2023, Aapo Väänänen, Waters Finland).
	Most of the chemicals and laboratory items of different manufacturers were purchased from either **VWR, Merck, or Fischer Scientific of Finland.**
	Dr. Ehrenstorfer GmbH is part of **LGC standards GmbH** (Wesel, Germany).
	Riedel de Haen and Fluka are part of **Honeywell specialty Chemicals Seelze GmbH** (Seelze, Germany).

## Introduction

Glyphosate [N-(phosphonomethyl) glycine] (GLY) has been one of the world's most widely applied herbicides since it came onto the market in 1974 [1], [2], [3]. It is a broad-spectrum, nonselective, post-emergence herbicide. Its major degradation product, aminomethylphosphonic acid (AMPA), is found in plants, water, and soil [4]. GLY consists of three polar functional groups (phosphonomethyl, amine, and carboxymethyl groups), and this makes it an ionic compound (log KOW=−3.20), very soluble in water, and a highly sorptive substance [5].

Determination of GLY and AMPA is possible with a reverse phase column by liquid chromatography–mass spectrometry (LC-MS/MS), when derivatisation is used. According to Raina-Fulton [6], half the articles reported determination of GLY and AMPA with 9-fluorenylmethyl chloroformate (FMOC) derivatives by LC-MS/MS with a positive ionisation (ESI+) mode. Sensitivity is then two times higher than by negative ionisation (ESI) mode and a lower interference signal has been detected. Ibañez et al. [4] reported 70–120% recovery with the limit of quantification 0.05 mg kg^−1^ for both GLY and AMPA with extraction of 0.6 M potassium hydroxide (KOH) solution and FMOC derivatisation utilising LC-MS/MS. Without derivatisation, either HILIC or ion chromatographic columns were used by LC-MS/MS run in negative ionisation (ESI) mode [6].

Autio et al. [7] found that adsorption of GLY to Finnish agricultural soils was high, and sorption was not found to be correlated with measured soil properties like organic carbon content, pH, and clay%. However, increasing soil phosphorus status decreases GLY adsorption and increases its desorption [8]. The environmental fate of GLY and AMPA were studied in field experiments conducted in different soil types [9,10]. In these field studies, GLY sorption was high, and soil phosphorus did not hinder sorption. The high sorption of GLY is linked to a decreased degradation rate and prolonged persistence in soil due to decreased microbe bioavailability [11,12]. Moreover, high sorption is often associated with poorer recoveries in residue analysis. Both these assumptions seemed to be true in the case of GLY in the Finnish field experiments.

Laitinen et al. [9] extracted GLY and AMPA from soil samples with 0.1 M KOH, cleaned up with methylene chloride extraction and cation exchange, and detected as their post-column o-phthalaldehyde (OPA) derivatives by a high-performance liquid chromatography and fluorescence detector (HPLC-FLD). The recovery of GLY and AMPA was studied in clay and sandy loam soil in two depths (0–28 cm and 28–50 cm). The recovery of GLY varied between the tested soil samples from 35± 8% to 59 ± 5%, being lowest on the clay soil site in plough depth (0–28 cm) and highest in sandy soil in a layer of 28–50 cm. Respectively, the values for AMPA were 46± 9% and 75 ± 7% [9]. The main reasons for the poor recoveries were a) a weak extraction solvent and b) multiphase long extraction with several clean-up phases. The poor recoveries increased the uncertainty of the interpretation of the results, and a more effective and more sensitive new method was needed for newer GLY fate studies in Finnish fields [13].

***The new method for quantification GLY and AMPA residues in clay soil in brief***

Potassium hydroxide solution (0.6 M) was chosen as the extraction solvent as by Ibañez et al. [4] because the previously used 0.1 M potassium hydroxide solution was insufficiently effective. The post-column HPLC-FLD residue method used by Laitinen et al. [9] was replaced by the multiple reaction technique (MRM) with ultra-high performance liquid chromatography–tandem mass spectrometry (UHPLC-MS/MS) [4]. However, several modifications were made in sample pre-treatment, calibration, use of internal standards, FMOC derivatisation and UHPLC-gradient.

Most of the published GLY and AMPA analysis of soil are based on air-dried soil extraction [e.g. 4,14,15], but we used fresh soil like Laitinen et al. [9,16]. Fresh soil was used to avoid unwanted processes like degradation during air-drying. At least three subsamples of each soil sample were analysed. In the event of high variation between the subsamples, two or three extra analyses were made. A soil sample was extracted twice, and no dilution or fortification of extract were not done.

Earlier experiences about analysing GLY and AMPA residues in leaching water samples showed that UHPLC-grade water in calibration samples suppressed MS signal of GLY-FMOC and AMPA-FMOC compared to MS-signal in natural leaching water samples (unpublished results). So, a multipoint matrix-matched calibration for quantification was used to minimise either possible suppressive or enhancing matrix effects. The calibration curve samples were pre-treated identically with the research samples.

The ^13^C,^15^N-analogies of GLY and AMPA were used as internal standards (IS) (Martin Ferencik, personal communication by email, 9 January 2013; unreferenced), whereas Ibañez et al. [4] used only GLY (1,2–^13^C,^15^N) for both compounds. Working solutions of ISs (WSIS) were added to the weighed soil sample before the extraction solvent because of the well-known strong adsorption of GLY in Finnish soil [7,8], whereas Ibañez et al. [4] added IS just before derivatisation with FMOC—Cl overnight. Hanke et al. [17] mentioned several derivatisation times, but we used 30 min at room temperature, which was used with leaching water and its solid matrix samples (unpublished results), previously with adsorption and desorption research [7,8], and originally by Sancho et al. [18] to avoid interfering FMOC derivatives during LC-MS/MS measurements [17]. The FMOC derivatives of the compounds were separated by a basic gradient UHPLC run as in Hanke et al. [17] and by Martin Ferencik (personal communication by email, 9 January 2013; unreferenced).

## Method details

### Soil samples

The soil sample (silty clay) for matrix-matched calibration was taken from environmental fallow, Kokemäki, Satakunta), where no herbicides were used for over 23 years. The soil samples for method development and validation were taken from the Kotkanoja experimental field, Jokioinen (60°49′N, 23°30′E), classified as Protovertic Luvisol (Clayic, Cutanic) and representing heavy clay soil [19], and from the old field samples collected in the field experiment (Perniö) conducted in clay soil [7,9] for method development and validation. Two additional topsoil samples (loam and sandy clay with a high content of organic matter) from farmers’ fields in Southwest Finland [(Janakkala and Forssa), taken in 2016, were used as reference samples for the method's revalidation during 2021. More information on soils is given in supplementary material (Table SM).

### Chemicals and reagents

GLY, GLY (1,2–^13^C,^15^N) (internal standard, IS1), AMPA, and AMPA (^13^C,^15^N) (internal standard, IS2) were purchased from Dr. Ehrenstorfer GmbH (Augsburg, Germany). Glufosinate-ammonium (GLUF) (Riedel de Haen, Buchs SG, Switzerland) was used as the method's recovery standard. The other reagents (Manufacturer) were disodium tetraborate decahydrate, methylene chloride, and hydrochloric acid (37–38%, 6 M) (J.T Baker, Deventer, the Netherlands), 9-fluorenylmethyl cloroformate (≥ 99.0%) (FMOC—Cl, Fluka, Switzerland), potassium hydroxide (KOH), acetonitrile (99.9%) and methanol (99.9%) (Prolabo, Leuven, Belgium), ammonium hydroxide (25%), UHPLC-MS-grade methanol (99.99%, Fischer Scientific, Loughborough, UK), and ammonium acetate (98%, Merck, Darmstadt, Germany). UHPLC-grade water was produced either by Siemens Ultra Clear Water (Siemens AG, Barsbuttel, Germany) or Merck MilliQ (Millipore, Billerica, MA, USA) systems.

### Laboratory equipment

All the standards’ stock and working solutions were prepared in UHPLC-grade water and in polypropylene (PP) vessels to avoid the adsorption of the standards on the surface of the glassware. Centrifuge tubes (PP) of 50 mL with screw caps were used to extract and neutralise extracts before derivatisation. FinnPipettes with PP tips were used for accurate pipetting. A Kipp pipette (25 mL) was used to add an extraction solution to the soil samples. Graduated glass test tubes (5 mL) were used in the FMOC derivatisation phase. Pasteur pipettes (VWR, 150 mL) were used for pH adjustment and spurtling with methylene chloride.

Analytical balance (Mettler AE 160, Columbus, Ohio, USA) was used to weigh the solid standards. Teflon spatulas were used for the weighing of soil samples with a top loading balance (Mettler PC 180, Columbus, Ohio, USA). The desired pH-values were checked with a pH indicator test strip (VWR, Finland). An ultrasonic bath (Branson 3510, Danbury, Connecticut, USA) and horizontal shaker (Heidolph Promax 2020, Schwabach, Germany) were used for the extraction of the soil samples. Supernatants were separated from soils by centrifugation (Heraeus Multifuge 4KR, Hanau, Germany). All the samples were filtrated with a 0.2 µm GHP filter (Pall Corporation, Ann arbor, MI, USA) and 5 mL PP syringes (Henk-Sass, Wolf GmbH, Tuttlingen, Germany) into sample bottles with screw caps (VWR, Finland) for a UHPLC-MS/MS run. The compounds’ FMOC derivatives were originally separated with Acquity UPLC BEH C18 1.7 µm-columns: VanQuard pre-column (2.1 × 5 mm) connected with an analytical column (2.1 × 100 mm) (Waters Corporation, Milford, MA, USA) but was replaced in 2021 by Acquity Premier UPLC BEH C18 1.7 µm-columns: VanQuard pre-column (2.1 × 5 mm) connected with an analytical column (2.1 × 100 mm) (Waters Corporation, Milford, MA, USA).

### Stock solutions of standards

Stock standard solutions (100 µg mL^1^) of GLY, AMPA, and GLUF were prepared by dissolving 5 ± 0.01 mg of a standard into UHPLC-grade water in a 50 mL volumetric flask (PP). An ultrasonic bath was used for dissolving.

Stock solutions of internal standard GLY (1,2–^13^C,^15^N) (100 µg mL^−1^, 1.1 mL) and AMPA (^13^C,^15^N) (100 µg mL^−1^, 1.1 mL) were purchased in amber glass ampoules.

**Working solutions of standards (WS)**

Three working solutions (WS) of standards were made in UHPLC-grade water:

WS1 (10 µg mL^−1^): 5 × 1000 µL of stock solutions of GLY, AMPA, and GLU were transferred into a 50 mL volumetric flask (PP), filled to the mark, and mixed well.

WS2 (1.0 µg mL^−1^): 1000 µL of stock solutions of GLY, AMPA, and GLU were transferred into a 100 mL volumetric flask (PP), filled to the mark, and mixed well.

WS3 (0.1 µg mL^−1^): 500 µL of WS1 was transferred into a 50 mL volumetric flask (PP), filled to the mark, and mixed well.

**Working solutions of internal standards (WSIS)**

WSIS1 (10 µg mL^−1^): 1.1 ml of GLY (1,2–^13^C,^15^N) (100 µg mL^−1^) was diluted with 9.9 mL of UHPLC-grade water in a PP test tube with a screw cap.

WSIS2 (10 µg mL^−1^): 1.1 mL 13C,15N-AMPA (100 µg mL^−1^) was diluted with 9.9 mL of UHPLC-grade water in a PP test tube with a screw cap.

**A recovery standard solution of GLUF** (0.5 µg mL^−1^) was prepared by transferring 250 µL of GLUF stock solution into a 50 mL volumetric flask (PP), filled to the mark, and mixed well.

### Other reagents

***6*** ***M KOH solution: stock solution for extraction solvent***

KOH grains (336.7 g) were weighed and poured with a funnel into a 1000 mL glass volumetric flask. The grains were dissolved in UHPLC-grade water, and the flask was cooled in a cold water bath at the same time. After the solution was cooled, the flask was filled to the mark with water and mixed well.

***0.6*** ***M KOH solution: extraction solution***

100 mL of 6 M KOH solution was measured with measuring glass into a 1000 mL volumetric flask, filled to the mark with water, and mixed well.

***6*** ***M and 0.6*** ***M hydrochloric acid in water: for neutralisation of extract***

0.6 M HCl: 10 mL strong hydrochloric acid (6 M) was diluted with water in a volumetric flask (100 mL), filled to the mark, and mixed well

***0.1*** ***M Disodium tetraborate decahydrate: buffer for derivatisation reaction***

*3.8* *g of disodium tetraborate decahydrate was weighed into a 100* *mL glass volumetric flask and* dissolved in water with sonication in a hot water bath. After dissolving and cooling, the flask was filled to the mark with water and mixed well. The solution was stored in a glass container at room temperature.

***FMOC—CL in acetonitrile (12*** ***mg mL^−1^): derivatisation reagent***

600 mg of FMOC—CL was weighed and dissolved in a 50 mL glass volumetric flask in acetonitrile and mixed well. The solution was stored in a glass container in a refrigerator.

### UHPLC gradient solutions

A basic UHPLC gradient was chosen to separate the FMOC derivatives of the compounds according to Martin Ferencik (personal communication by email, 9 January 2013; unreferenced):

*Channel A1:* 10 mL of 0.5 M ammonium acetate solution, 5 mL of UHPLC-grade methanol, and 2.5 mL of ammonium hydroxide were mixed in a test tube (pH 10) and filtrated through 0.2 µm GHP into a 1000 mL volumetric flask. 20 mL of UHPLC-grade methanol was then added to stop bacterial growth and finally filled to the mark with UHPLC-grade water (final 5 mM ammonium acetate solution in water, 2.5% methanol, pH 9).

*Channel B1:* 5 mL of 0.5 M ammonium acetate solution, 2.5 mL of UHPLC-grade methanol, and 2 mL of ammonium hydroxide were mixed in a test tube (pH 10) and filtered through 0.2 µm GHP into a 500 mL volumetric flask and filled to the mark with UHPLC-grade methanol (final 5 mM ammonium acetate solution in methanol, pH 8).

### Matrix‐matched calibration

Three grams (3.0 *g* ± 0.5 g) of fresh GLY-, AMPA-, and GLUF-free clay soil (from Kokemäki, see Table SM) was weighed into 10 PP test tubes (50 mL) for matrix-matched calibration (STD 0 + 9 levels). The WS of the standards and UHPLC-grade water were added to them according to Table 1 so the matrix amount and water volume were the same in the calibration standards as in the research samples. Concentrations have been given both as ng mL^−1^and mg kg^−1^ (in fresh soil) in the table. The addition of WSISs and the extraction is described in the **Sample extraction** chapter.

**Table 1:** The volumes of each working solution (WS3, WS2, and WS1) and UHPLC-grade water (H_2_O) in 3 g of fresh soil for the matrix-matched calibration. Concentrations are given for both ng mL^−1^ and mg kg^−1^.

Cal	ng mL^−1^	WS3 (µL)	WS2 (µL)	WS1 (µL)	H_2_O (µL)	mg kg^−1^*
0	0.0	–	–	–	1000	0.0
1	0.5	250	–	–	750	0.01
2	1.0	500	–	–	500	0.02
3	2.0	1000	–	–	–	0.03
4	5.0	–	250	–	750	0.08
5	10	–	500	–	500	0.16
6	20	–	1000	–	–	0.33
7	50	–	–	250	750	0.82
8	100	–	–	500	500	1.65
9	200	–	–	1000	–	3.31

⁎ in fresh soil.

### Sample extraction

Three replicates of each soil sample (3.0 *g* ± 0.5 g) were weighed for analysis because of the heterogeneity of the real clay soil samples. A recovery standard solution of GLUF (1000 µL, 0.5 µg) was added only to the research samples, not in matrix-matched calibration samples. WSISs (both WSIS1 and WSIS2, 50 µL, 0.5 µg) were added to the soil (both matrix-matched calibration and research samples) before the extraction solvent. Each soil sample was extracted twice with a 25 mL 0.6 M KOH solution with horizontal shaking (15 min, 220 min^−1^), followed by sonication (60 min). A supernatant was separated from the soil by centrifugation (3500 × g, 15 min). Aliquots (5 mL) of both 25 mL of extracts were combined, mixed well, and neutralised with 6 M and 0.6 M hydrochloric acid solution (pH 8 ± 0.5). The resulting sediment was separated from the neutralised extract by centrifugation (3500 × g, 10 min).

### Derivatisation of calibrants and samples

One (1) mL of neutralised extract, either calibrant or research sample, and 0.5 mL of disodium tetraborate buffer (0.1 M in water) were transferred into a glass test tube (5 ml) and mixed well, with pH adjusted to 9–10[18]. Then 1 mL of FMOC—Cl in acetonitrile (12 mg mL^−1^) was added and mixed well [4], and incubation was allowed at room temperature for 30 min [7,^8^,18]. The reaction was stopped by adding 2.5 mL methylene chloride (DCM). Extra FMOC—Cl and acetonitrile were washed into the DCM phase by spurtling with a Pasteur pipette, whereas Sancho et al. used ethyl acetate for this [18]. The phases were settled for 10 min, better separation was facilitated by centrifugation (3500 × g, 10 min), and the upper water phase was filtered with a 0.2 µm GHP filter and syringe into a UHPLC vial.

### Instrumentation

Ultra-high-performance liquid-chromatography connected with tandem mass-spectrometry (Waters Acquity UPLC Xevo TQ MS) was used to identify and quantify the compounds. The FMOC derivatives of the compounds were separated with an Acquity UPLC BEH C18 column. The column temperature was 40 °C, the flow in the gradient run was 0.3 mL min^−1^, and the injection volume was 5 µL. The Basic UHPLC gradient (Table 2) was used to separate the FMOC derivatives of the compounds.

**Table 2:** Ultra-high performance liquid chromatography (UHPLC) gradient for separation of compounds. The total run time was 12 min (min).

Time (min)	Flow (mL min^−1^)	A1 (%)	B1 (%)	Curve
0.0	0.3	95	5.0	6
3.0	0.3	70	30	6
6.0	0.3	50	50	6
7.0	0.3	0	100	6
8.0	0.3	0	100	6
9.0	0.3	95	5.0	11

The compounds were identified and quantified with an internal standard method and MRM technique with UHPLC-MS/MS. Nitrogen was used as a desolvation gas and argon as a collision gas [4]. The capillary voltage was 3.0 kV in electrospray ionisation in positive mode (ESI+). The desolvation gas flow was 1000 L/h, while the cone gas flow was 19 L/h. The desolvation temperature was 600 °C. The IntelliStart of the instrument was used to search for MRM reactions for GLY-, AMPA-, and GLUF-FMOC (Table 3). MRM reactions for the ISs were either three or two units higher than for GLY and AMPA, but the retention times (RT) were the same. The first MRM of each compound was used for quantification, and the second for confirmation.

**Table 3:** Multiple reaction monitoring (MRM) settings of the fluorenyl methyl chloroformate (FMOC) derivatives of glyphosate (GLY), aminomethylphosphonic acid (AMPA), their 1^3^C,^1^^5^N analogies and glufosinate-ammonium (GLUF). The precursor ion was molecular ion + proton [M+H]^+^. The product ion was produced by the given collision energy.

Compound	Precursor ion (m/z)	Product ion (m/z)	Dwell (s)	Cone (V)	Collision (V)	RT (min)
GLY-FMOC	392.1	87.9	0.019	20	16	3.5
	392.1	214	0.019	20	9	3.5
1,2-^1^^3^C,^15^N -GLY-FMOC 1	395.1	90.99	0.019	20	16	3.5
	395.1	217	0.019	20	12	3.5
AMPA-FMOC	334.1	156	0.019	20	8	5.8
	334.1	112	0.019	20	13	5.8
^1^^3^C,^15^N -AMPA-FMOC 2	336.1	158	0.019	20	8	5.8
	336.1	114	0.019	20	12	5.8
GLUF-FMOC 3	404.1	136	0.019	20	20	5.6
	404.1	182	0.019	20	16	5.6

1 Internal standard for GLY-FMOC

2 Internal standard for AMPA-FMOC

3 Recovery standard of the method

## Method validation

### Method performance study

The results were not corrected for recovery because a) research soil samples and matrix matched calibrants were handled similarly, and b) multipoint matrix-matched calibration and c) an internal standard (IS) method were used. A single analysis was monitored by the recovery of GLUF, which was also quantified with multipoint matrix-matched calibration, but with an external standard calibration curve. Calibration curve samples were run from the lowest to highest levels, followed by a solvent injection before real sample injections to prevent any carryover from calibrants. The calibration curve was repeated at the end of the sample list. Standard 20 ng mL^−1^ was run after every 9 samples as a control sample, followed by a solvent injection.

### Limit of detection and limit of quantification

The lowest calibration level of the method was 0.5 ng mL^−1^ (Cal1: 0.01 mg kg^−1^ in fresh soil). Lower concentrations could be detected, but the signal-to-noise value (S/N) was not usually satisfied in those cases. Average concentrations and standard deviations (SD) were calculated with 40 AMPA results and 63 GLY results under 0.5 ng mL^−1^ in research samples from the Kotkanoja leaching field. The limit of detection (LOD) was calculated as [Average concentration + (3 × SD)], and the limit of quantification (LOQ) was [Average concentration + (6.5 × SD)] [20]. The LOD was 0.01 mg kg^−1^ (Cal1), and the LOQ was 0.02 mg kg^−1^ (Cal2) for both compounds in fresh soil. The LOQ was 0.02 mg kg^−1^ for GLY and 0.03 mg kg^−1^ for AMPA in dry matter. The lowest dry matter in these samples was 65%. The results of these calculation have been given in Table 4.

**Table 4:** The limit of detection (LOD) and the limit of quantification (LOQ) as mg kg^−1^, both (a) in fresh and (b) in dry soil, as repeatability and producibility of the method (%) for aminomethylphosphonic acid (AMPA) and glyphosate (GLY).

Compound	LOD (mg kg ^−1^) a	LOQ (mg kg ^−1^) a	LOQ (mg kg^−1^) b	Repeatability (%)	Reproducibility (%)
AMPA	0.01	0.02	0.03	19.5	23.6
GLY	0.01	0.02	0.02	25.0	28.1

a in fresh soil.

b in dry soil.

### Repeatability and reproducibility

The repeatability of the AMPA and GLY analysis was followed by at least three parallel analyses of samples. Altogether, the relative standard deviations (RSDs) of AMPA and GLY concentrations in 20 soil samples were used for the repeatability calculation. Eight of the samples were analysed by several laboratory assistants, so the final number of RSDs (n) was 38 for repeatability. The RSDs of AMPA and GLY in the eight soil samples were used for the reproducibility calculation (*n* = 8).

$$\text{Repeatability}\;\%\;\left(\text{or}\;\text{reproducibility}\;\%\right)=\sqrt{(\sum \left({RSD)}^{2}\right)/n}$$

The results of the repeatability and reproducibility calculations are given in Table 4. The repeatability was slightly better than the reproducibility. The uncertainty of results linked to the repeatability and reproducibility was less than 30% for both compounds.

### Matrix effect and linearity

The validation was completed with several matrix matched calibrations in GLY-, AMPA-, and GLUF-free soil: both 0–10 cm (from Kokemäki; clay soil, see Table SM) and 35–55 cm soil depth (from the Kotkanoja, clay soil, see Table SM) were used (Table 5). Spiked herbicides, concentrations of WSs given in Table 1, were extracted a) immediately after adding (+25 °C), b) after 10 days incubation in a refrigerator (+4 °C), and c) after 10 days in a freezer (−25 °C). Each calibration curve samples were quantified with the other curves. Irreversible adsorption of analytes did not occur, and GLY was not degraded to AMPA. The slopes of AMPA and GLY were near 1 because the internal standard (IS) method was used for them. The y-axis interception of AMPA and GLY was also near the origin, which mean the matrix effect could be controlled using own IS for both AMPA and GLY and adding WSISs at the beginning of the sample pre-treatment. The correlation (R^2^) of each curve was ≥ 0.99 (except one of GLUF R^2^ = 0.983), which means calibration is linear in the studied concentration range (0.5–200 ng mL^−1^ or 0.01–3.31 mg kg^−1^ in fresh soil), although LOQ in the real research samples was 0.02 mg kg^−1^ in fresh soil.

**Table 5:** Comparison of matrix-matched calibration of aminomethylphosphonic acid (AMPA), glyphosate (GLY) and glufosinate-ammonium (GLUF) in two different soils (sampling place and depth), which were extracted (a) immediately (+25 °C) and after a 10-day period in either (b) a refrigerator (+4 °C) or (c) a freezer (−25 °C).

Compound	Calibration curve	Slope Kokemäki	Slope Kotkanoja	Intercept Kokemäki	Intercept Kotkanoja	R^2^ Kokemäki	R^2^ Kotkanoja
AMPA	0 day/+25 °C a	0.989	0.914	0.204	0.166	0.997	0.998
	10 days/+4 °C b	0.933	0.937	0.307	0.148	0999	0.997
	10 days/−25 °C c	0.890	0.907	0.676	0.210	0.998	0.998
	**Average**	**0.937**	**0.919**	**0.396**	**0.175**	**0.998**	**0.998**
GLY	0 day/+25 °C a	0.985	0.914	−0.101	0.166	0.996	0.998
	10 days/+4 °C b	0.986	0.901	−0.748	0.148	0.992	0.998
	10 days/−25 °C c	0.886	0.960	−0.223	0.117	0.999	0.995
	**Average**	**0.952**	**0.925**	**−0.357**	**0.144**	**0.995**	**0.997**
GLUF	0 day/+25 °C a	373.7	454.1	−81.34	−115.7	0.983	0.996
	10 days/+4 °C b	334.3	375.2	−120.8	−352.8	0.997	0.988
	10 days/−25 °C c	343.4	437.6	43.47	−10.10	0.997	0.999
	**Average**	**350.4**	**422.3**	**−52.90**	**−159.5**	**0.992**	**0.994**

a extracted immediately (+25 °C).

b extracted after 10-day period in a refrigerator (+4 °C).

c extracted after 10-day period in a freezer (−25 °C).

The six calibration curves presented in Table 5 were used for the quantification of seven soil samples, sampled at six different depths. Average concentrations (mg kg^−1^) and standard deviations (SD) are given in Table 6. The recovery of GLUF was 80–119% (theoretical recovery for concentration of 0.16 mg kg^−1^ in fresh soil), which shows the extraction was successful. Concentrations of AMPA varied from <LOQ to 3 mg kg^−1^; concentrations of GLY varied from < LOQ to 1 mg kg^−1^ in these samples. The minor standard deviation of AMPA and GLY concentrations showed that the 0.6 M KOH was sufficiently effective to extract all the analytes from the different soil layers.

**Table 6:** Average concentrations (mg kg^−1^) and standard deviations of aminomethylphosphonic acid (AMPA), glyphosate (GLY), and glufosinate-ammonium (GLUF) in seven soil samples taken from Kotkanoja (see supplementary material, Table SM), quantified with six different calibration curves presented in Table 5.

Sample code 1	Depth (cm)	AMPA ± SD 2	GLY ± SD 2	GLUF ± SD 2
2012–287–1 B	0–2.5	2.96 ± 0.10	0.99 ± 0.05	0.15 ± 0.02
2012–287–4 B	2.5–5.0	1.11 ± 0.04	0.25 ± 0.01	0.15 ± 0.02
2012–287–5 B	5.0–10	0.50 ± 0.02	0.13 ± 0.01	0.16 ± 0.02
2012–287–8 B	2.5–5.0	0.94 ± 0.03	0.37 ± 0.02	0.16 ± 0.02
2011–261–46 A	10–25	0.12 ± 0.004	0.04 ± 0.01	0.16 ± 0.02
2011–261–47 A	25–35	< LOQ	< LOQ	0.17 ± 0.02
2011–261–48 A	35–55	< LOQ	< LOQ	0.17 ± 0.02

1 A or B mean subsample of research soil.

2 = mg kg^−1^ in fresh soil.

### The comparison of the old and new method

The effectivity of the new method was able to test the analysis of some old soil samples in 2015. These had been collected in earlier glyphosate fate studies [9] and had been kept frozen after analysis in 2000 and recovery tests in 2004. GLY and AMPA residues were extracted with 0.1 M KOH solution and determined as OPA post-column derivatives by HPLC-FLD at that time. The results of both determinations are presented in Table 7 below. It shows the recoveries in real residue samples were even lower in 2000 than in the recovery test in 2004 (GLY 35–59% and AMPA 46–75%) [9], although the results of the background samples were similar. The interaction of GLY and AMPA in recovery test soil was less strong than adsorption in the real residue soil samples. Additionally, there was an extra loss of recovery during the clean-up phases of the old method.

**Table 7:** Glyphosate (GLY) and aminomethylphosphonic acid (AMPA) residues in three old clay (SA) samples taken from Perniö (see supplementary material, Table SM) using old (2000) and new (2015) methods.

Sample code	Sampling	Depht	Compound	2000 2	2015 3
				mg kg^−1^	mg kg^−1^ ± SD
SA.1.99.4 1	Spring 1999	0–28 cm	GLY	< 0.05	0.04 ± 0.01
			AMPA	0.05	0.04 ± < 0.01
SA.18.00.GLY.1	25.7.2000	0–28 cm	GLY	0.51	3.27 ± 0.31
			AMPA	0.09	0.44 ± 0.05
SA.19.00.GLY.1	10.8.2000	0–28 cm	GLY	1.75	6.96 ± 0.64
			AMPA	0.30	0.92 ± 0.06

1 background sample, used also for recovery test in 2004.

2 0.1 M KOH extraction [9].

3 0.6 M KOH extraction.

### Revalidation of the method

If the analytical method has not been used for a while, it must revalidate. Two topsoil samples were first analysed as research samples in 2017 [unpublished results] and again as reference samples in 2021. The samples were kept frozen before and between the analyses. The number of analysed subsamples were three (*n* = 3) in 2017 and four (*n* = 4) in 2021. The average concentrations (AV) and their standard deviation (SD) in both years and the AV and SD of all 7 subsamples are given in Table 8. The quantified concentration of AMPA was quite stable both years, but average concentration of GLY varied more. The average results of both AMPA and GLY were at the same concentration level. It can be speculated whether freezing and thawing increased the extractable proportion of the herbicides analogically to the observations made by e.g. Holten et al.in minilysimeter study [21] and by Siimes et al. in field study about increased herbicide leaching in partially frozen soil [22]. However, these results confirmed the original repeatability and reproducibility for GLY, even better values could be achieved for AMPA.

**Table 8:** Average concentrations (mg kg^−1^) and standard deviations (SD) of glyphosate (GLY) and aminomethylphosphonic acid (AMPA) residues in two topsoil samples (Janakkala and Forssa, see supplementary material, Table SM) by new method in 2017 and 2021. The number of analysed subsamples (n) is given. All (*n* = 7) mean combined result of both years.

Sample code	Sampling	Depht	Compound	2017 (*n* = 3) 1	2021 (*n* = 4) 2	All (*n* = 7)
	(Location)			mg kg^−1^ ± SD	mg kg^−1^ ± SD	mg kg^−1^ ± SD
16–243–23	19.5.2016	0–2.5 cm	GLY	1.6 ± 0.5	2.3 ± 0.6	2.0 ± 0.7
	(Janakkala)		AMPA	2.3 ± 0.2	2.4 ± 0.2	2.4 ± 0.2
16–405–5	23.9.2016	0–2.5 cm	GLY	2.6 ± 0.1	3.4 ± 0.3	3.0 ± 0.5
	(Forssa)		AMPA	1.9 ± 0.2	2.0 ± 0.2	1.9 ± 0.2

1 Acquity UPLC BEH C18 column was used for separation of analytes.

2 Acquity Premier BEH C18 column was used for separation of analytes.

## Conclusion

This article has presented an effective new method for the determination of GLY and AMPA residues in Finnish soils. The most important improvements compared to previous methods were a) using 0.6 M KOH as an extraction solution, b) using ^13^C,^15^N- analogies of GLY and AMPA as internal standards and adding them at the beginning of the sample pre-treatment, c) using multipoint matrix-matched calibration, which samples were pre-treated identically with the research samples, and d) identification and quantification of the FMOC derivatives of all analytes by MRM-UHPLC-MS/MS technique.

It was challenging to find a GLY and AMPA free soil for matrix calibration for the Kotkanoja experimental field soil samples because there was a long history of using GLY [19]. Even with organic farming, there may be at least interference AMPA residues if GLY products has been used in the past. However, environmental fallows have often not been involved in intensive farming for several years, and no herbicide treatments have been done there, which enabled a GLY- and AMPA-free soil for method development and calibration to be found.

The quality of ^13^C,^15^N- analogies of GLY and AMPA proved critical for quantification: the method development for soil residues was started with twice the volume of both ISs because the quality of ^13^C,^15^N-AMPA between different lots varied so greatly. Later, the change of the lots of the ISs did not weaken the quantification GLY and AMPA.

The use of 0.6 M KOH was sufficiently strong to release adsorbed GLY and AMPA from the soil when extraction was repeated. GLUF, which will not adsorb in Finnish soil [7], was practicable for following the effectiveness of extraction. Replacing the Acquity UPLC BEH C18 column with a Premier one improved the intensity of analytes and especially diminished their daily variation.

The internal standard method of GLY and AMPA with a multipoint matrix-matched calibration was the best way to control matrix effect in soil. The amount of soil matrix was similar in the calibration curve samples and in the research samples, when the pre-treatment was identical.

The method was originally developed and validated for clay soils in “The environmental risks of glyphosate use: Transport in clay soils and leaching to watercourses’’ research between 2011 and 2014 at MTT Agrifood Research Finland. Since 2015, it has also been utilised reliably in forest soil samples from Northern Savonia and field soil samples from Southwest Finland (both unpublished results) and in the ongoing “Occurrence of pesticide residues in agricultural soils in northern climate” research (unpublished results) at Natural Resources Institute (Luke).

## CRediT author statement

**Sari Rämö**: Method development, method validation, instrument operation, quality control, data handling, writing – original draft preparation, reviewing and editing. **Juho Välimäki**: Method development, method validation, sample pre-treatment, instrument operation, writing-reviewing. **Katri Siimes**: Writing-reviewing and editing. **Jaana Uusi-Kämppä**: Project management of “The environmental risks of glyphosate use: Transport in clay soils and leaching to watercourses’’ project and writing-reviewing and editing.

## Funding

This study was supported by the Development Fund for 10.13039/501100011056Agriculture and Forestry (MAKERA) [grant number 2693/312/2010]; Finland, the Marjatta and Eino Kolli Foundation, Vierumäki, FI [grant number 1073]; and 10.13039/501100013914Maa- ja vesitekniikan tuki ry. Helsinki, FI [grant number 30481].

## Declaration of Competing Interest

The authors declare that they have no known competing financial interests or personal relationships that could have appeared to influence the work reported in this paper.

## Data Availability

Data will be made available on request.

## Appendix Supplementary materials

Image, application 1
