# Supplementary material for: Determination of glyphosate and aminomethylphosphonic acid residues in Finnish soils by ultra‐high performance liquid chromatography–tandem mass spectrometry
Source: MethodsX. 2023 Sep 23;11:102397. doi: 10.1016/j.mex.2023.102397 (PMC10542195; doi:10.1016/j.mex.2023.102397)
Supplement: Supplementary file 1 [file mmc1.docx]

Table SM. Sampling depth, soil type, particle size distribution, pH, organic carbon (OC), cation exchange capacity (CEC) [SM1] and bulk density (BD)

| **Site** | Depth  [cm] | Soil type | Clay  [%] | pH | OC  [%] | CEC  [cmol(+) L^-1^] | BD  [g L^-1^] |
| --- | --- | --- | --- | --- | --- | --- | --- |
| **Kokemäki**: environmental fallow | 0–10 | silty clay | n.a. | 5.9 | n.a. | 13 | n.a. |
| Jokioinen: **Kotkanoja** experimental field [SM2] | 0–24 | clay | 48 | 6.5 | 2.7 | 25^a^ => 30^b^ | 1198 |
| Jokioinen: **Kotkanoja** experimental field [SM2] | 24–32 | clay | 58 | 4.8 | 0.6 | 19^a^ => 24^b^ | 1245 |
| Jokioinen: **Kotkanoja** experimental field [SM2] | 32–56 | clay | 80 | 5.3 | 0.4 | 30^a^ => 37^b^ | 1240 |
| **Perniö**: previous glyphosate field studies in the site [SM3, SM4, SM5] | 0–30 | clay | 41 | 6.0 | 7.1 | n.a. | 1040 |
| **Janakkala**: a cultivated field | 0–2.5 | loam^c)^ | n.a. | 6.7 | 18.9 | 33 | 532 |
| **Forssa**: a cultivated field | 0–2.5 | sandy clay ^c)^ | na | 5.5 | 14.2 | 30 | 553 |

n.a.= not analysed

a) unit: cmol(+) kg^-1^;

b) calculated from (a) using BD

c) high organic matter content

References

[SM1] R. Niskanen, A. Jaakkola. Estimation of cation-exchange capacity in routine soil testing. Journal of Agricultural Science in Finland 58 (1986) pp. 1–7

[SM2] T. Peltovuori, R. Uusitalo, T. Kauppila, T. 2002. Phosphorus reserves and apparent phosphorus saturation in four weakly developed cultivated pedons. Geoderma 110 (2002) pp. 35–47

[SM3] S. Autio, K. Siimes, P. Laitinen, Sl Rämö, S. Oinonen, L. Eronen, Adsorption of sugar beet herbicides to Finnish soil. Chemosphere 55 (2004) pp.215-226. doi:10.1016/j.chemosphere.2003.10.015

[SM4] P. Laitinen, K. Siimes, S. Rämö, L. Jauhiainen, L. Eronen, S. Oinonen, H. Hartikainen, Effects of Soil Phosphorus Status on Environmental Risk Assessment of Glyphosate and Glufosinate-Ammonium. J. Environ. Qual. 37 (2008) pp. 830–838. [**https://doi.org/10.2134/jeq2007.0256**](https://doi.org/10.2134/jeq2007.0256)

[SM5] P. Laitinen, K. Siimes, L. Eronen, S. Rämö, L. Welling, S. Oinonen, L. Mattsoff, M. Ruohonen-Lehto, Fate of the herbicides glyphosate,glufosinate-ammonium, phenmedipham,ethofumesate and metamitron in twoFinnish arable soils Pest. Manag. Sci., 62 (2006) pp. 473-491. [**https://doi.org/10.1002/ps.1186**](https://doi.org/10.1002/ps.1186)

Loput voikin jo unohtaa..

Table x. Sampling depth, soil type, particle size distribution, pH, organic carbon (OC), cation exchange capacity (CEC) and bulk density (BD)

| ~~Table x. Sampling depth, soil type, particle size distribution, pH, organic carbon (OC), cation exchange capacity (CEC) and bulk density (BD)~~ | | | | | | | | | |
| --- | --- | --- | --- | --- | --- | --- | --- | --- | --- |
| Locality | Depth | Soil type | Particle size distribution | | | pH | OC | CEC ^[x3]^ | BD |
|  | cm |  | % | | |  | % | cmol(+) L^-1^ | g L^-1^ |
|  |  |  | clay  <0.002 mm | 0.002–0.02 mm | ~~0.02–0.2~~ |  |  |  |  |
| Kokemäki | 0–10 | silty clay | n.a. | n.a. | ~~n.a.~~ | 5.9 | n.a. | 13 | n.a. |
| Jokioinen^[x1, x2]^ | 0–20 | clay | 61 | 16 | ~~23~~ | 6.5 | 2.7 | 25.2 ^(a)^ | 1180 |
| Perniö (Laitinen et al. 2006; 2008) | 0–30 | clay | 41 | 24 | ~~35~~ | 6.0~~2~~ | 7.1 | n.a. | 1040 |
| Janakkala A (Laitinen et al. 2006, 2008) | 0–30 | sandy loam | 4 | ~~15~~ | ~~81~~ | 6.4 | 6.8 |  | 1160 |
| Janakkala | 0–2.5 | loam | n.a. | n.a. | ~~n.a.~~ | 6.7 | 18.9 | 33 | 532 |
| Janakkala | 2.5–25 | loam | n.a. | n.a. | ~~n.a.~~ | 5.6 | 18.4 | 21 | 501 |
| Forssa | 0–2.5 | sandy clay | n.a. | n.a. | ~~n.a.~~ | 5.5 | 14.2 | 30 | 553 |
| Forssa | 2.5–25 | sandy clay | n.a. | n.a. | ~~n.a.~~ | 5.6 | 16.8 | 29 | 562 |

(a) unit: cmol(+) kg^-1^

n.a.= not analysed

[x1] = Turtola, E., Alakukku, L., Uusitalo, R. & Kaseva, A. 2007. Surface runoff, subsurface drainflow and soil erosion as affected by tillage in a clayey Finnish soil. Agricultural and Food Science 16: 332–351.

[x2] = Peltovuori, T., Uusitalo, R. & Kauppila, T. 2002. Phosphorus reserves and apparent phosphorus saturation in four weakly developed cultivated pedons. Geoderma 110: 35–47.

[x3] = Niskanen, R. & Jaakkola, A. 1986. Estimation of cation-exchange capacity in routine soil testing. Journal of Agricultural Science in Finland 58: 1–7.

VÄLLISSÄ KUVAKAAPPAUKSIA JA LOPUSSA EHDOTUS TAULUKOKSI

SYTTY maat: Laitinen et al. 2006. Tilavuuspainomäärityksissä vähän erilainen syvyysjakauma.


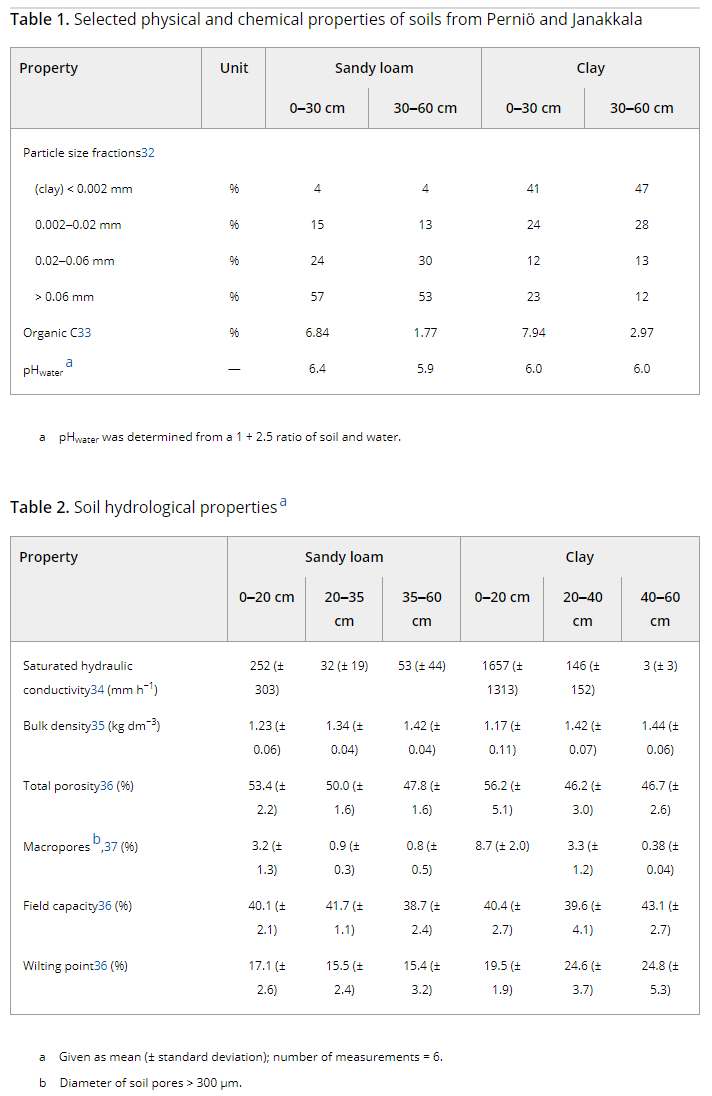


Tässä alla nää sorptiokokeissa käytetyt maat. Maista 1-10 tehtiin sorptioanalyysit kaikille 5 herkbisidille, maat 11- olivat mukana vain glyfosaatti ja glufosinaatti-ammoniumin sorptiokokeissa. Soil #7 ja 8 (Perniö) olivat Sytty-hankkeen koepeltoina Sokerijuurikkaan tutkimuslaitoksen lähellä. Ensin sinne valittiin toiset maat (muistaakseni maa #15), mutta niissä oli viljavuusfosfori liian suuri ja kenttäkokeen paikkaa vaihdettiin. Turengissa taas oli joku muu syy, että koepaikkaa vaihdettiin. Sytty-kokeiden jäämänäytteet otettiin pintakerroksesta (0-3 cm), 3 cm – muokkauskerroksen alareuna, jonka syvyys vähän vaihteli eri kerroilla. Ja sitten oli toki syvempiäkin näytteitä..


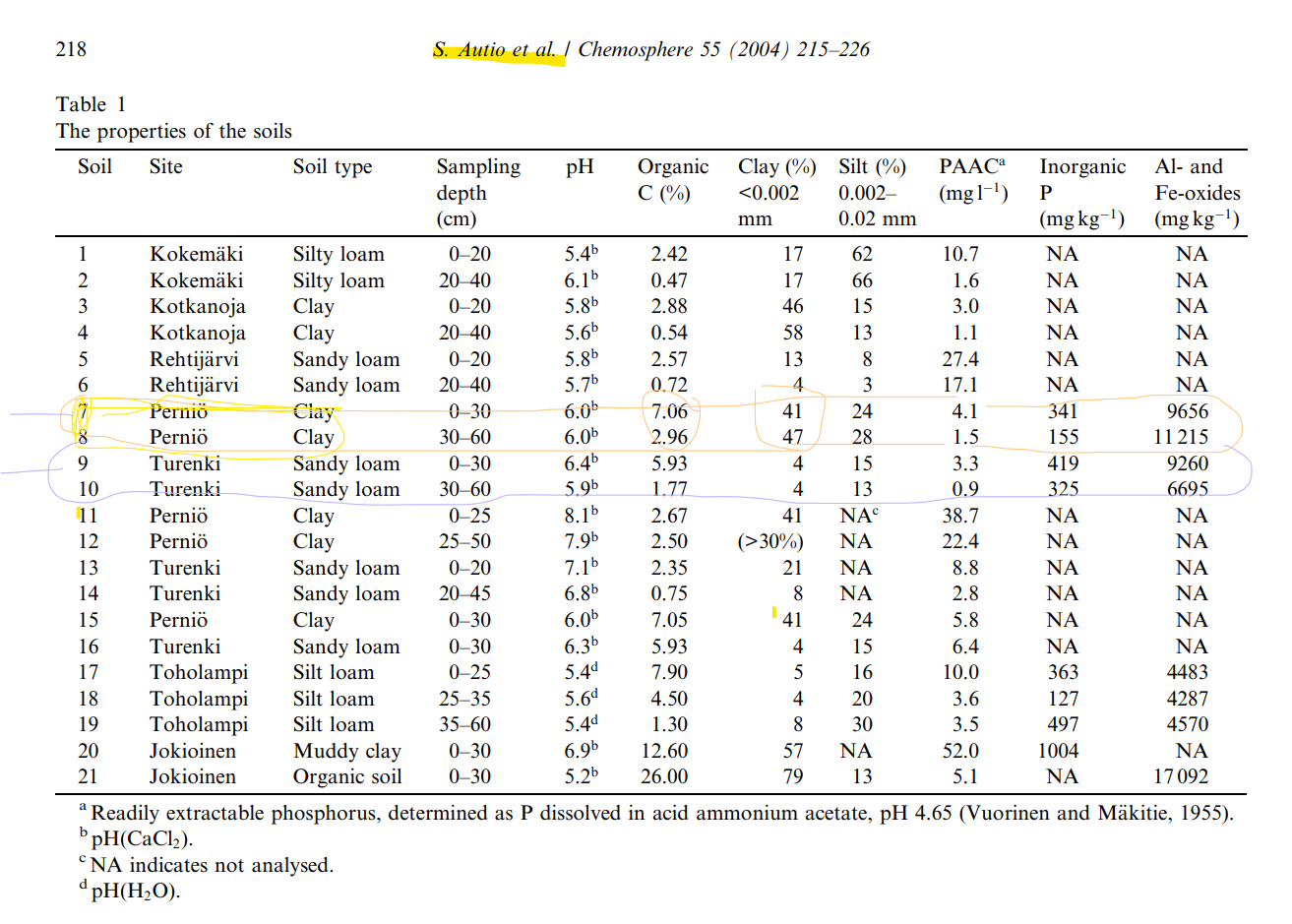


Tässä vielä SYTTY-kenttäkokeiden maat tarkemmin. Tässä määritetty mm. rauta- ja alumiinioksidit, epäorgaanisen fosforin fraktiot.

Lähde Laitinen et al. 2008. (Tätä varten teetettiin vielä noita uusia määrityksiä, vaikka osa tiedoista oli saatavillakin. Ja tuloksia väännettiin pitkään… yötä myöten useamman kerran)


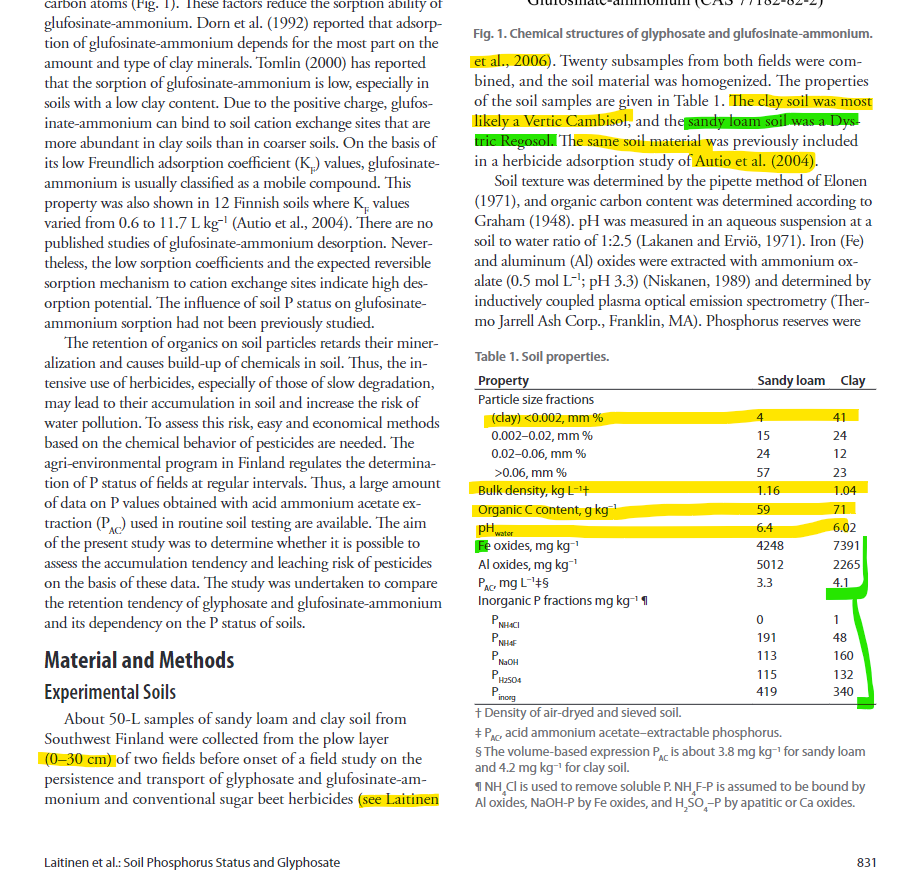


Muutamassa nyt taulukoidussa Glyfos2 maassa OC% on hyvin korkea. Alla kuvakaappaus luokittelusta. Jos orgaanisen aineksen osuus on yli 6 % pitäisi se tämän luokittelun mukaan ottaa jo huomioon nimessäkin. Esim. Glyfos2 viljelijämaat olisivat Organic mineral soil. tai Peaty loam.

“A conversion factor of 1.72 is used to convert organic matter to organic carbon”)


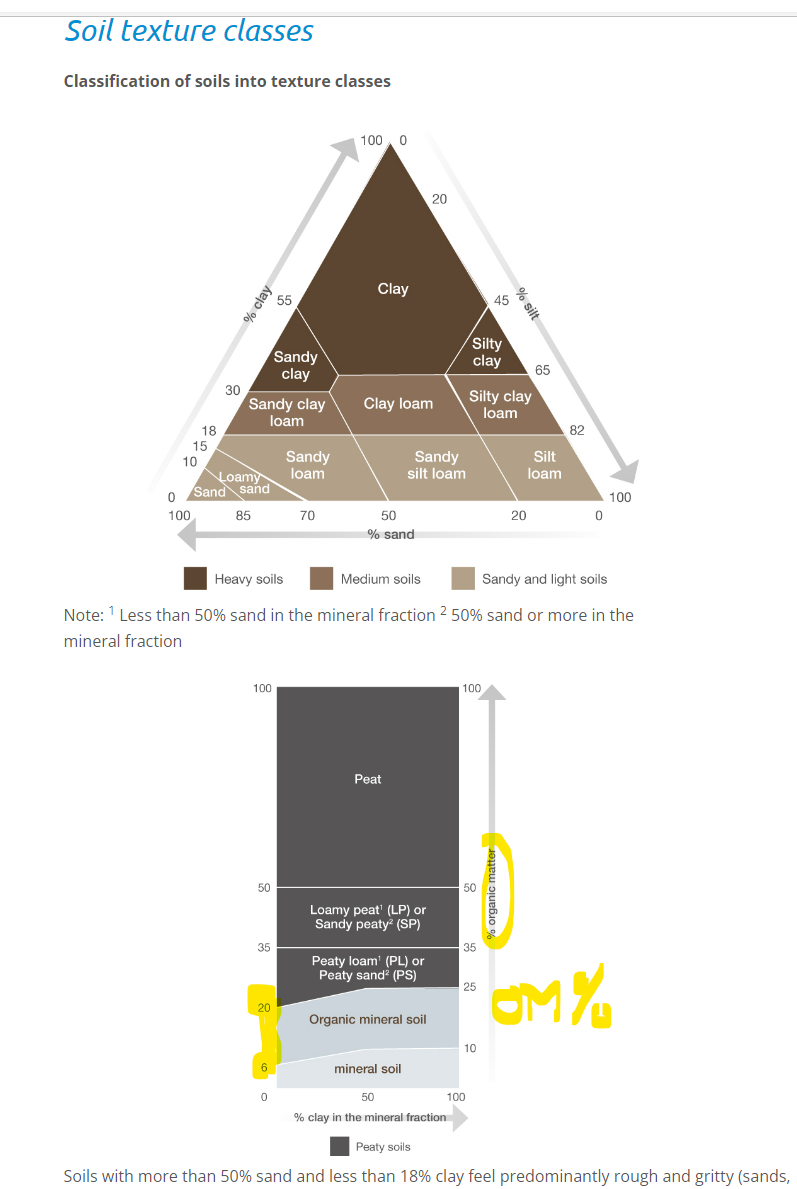


EHDOTUS: kommentteineen

Table x. Sampling depth, soil type, particle size distribution, pH, organic carbon (OC), cation exchange capacity (CEC)^VIITEX3^ and bulk density (BD)

| Locality | Depth | Soil texture type | Clay | pH | OC | OM | CEC ^[x3]^ | BD |
| --- | --- | --- | --- | --- | --- | --- | --- | --- |
|  | cm |  | % |  | % | % | cmol(+) L^-1^ | g L^-1^ |
| Kokemäki | 0–10 | silty clay | n.a. | 5.9 | n.a. |  | 13 | n.a. |
| Jokioinen^[x1, x2]^ | 0–20 | clay | 61 | 6.5 | 2.7 | 4,6 | xxxx  25.2 ^(a)^ | 1180 |
| Perniö | 0–30 | clay | 41 | 6.0 | 7.1 | 12,2 | n.a. | 1040 |
| Janakkala A | 0–30 | sandy loam | 4 | 6.4 | 6.8 |  | n.a |  |
| ~~Janakkala~~ | ~~0–2.5~~ | ~~loam~~ | ~~n.a.~~ | ~~6.7~~ | ~~18.9~~ | ~~32,5~~ | ~~33~~ | ~~532~~ |
| ~~Janakkala~~ | ~~2.5–25~~ | ~~loam~~ | ~~n.a.~~ | ~~5.6~~ | ~~18.4~~ | ~~31,6~~ | ~~21~~ | ~~501~~ |
| Janakkala | 0–25 | loam tai peaty loam | n.a. |  | 18.5 | 31,7 | 22 | 504 |
| ~~Forssa~~ | ~~0–2.5~~ | ~~sandy clay~~ | ~~n.a.~~ | ~~5.5~~ | ~~14.2~~ | ~~24,4~~ | ~~30~~ | ~~553~~ |
| ~~Forssa~~ | ~~2.5–25~~ | ~~sandy clay~~ | ~~n.a.~~ | ~~5.6~~ | ~~16.8~~ | ~~28,9~~ | ~~29~~ | ~~562~~ |
| Forssa | 0–25 | sandy clay tai peaty loam | na | 5.6 | 16.5 | 28 | 29 | 561 |
